# Screening of anti-dengue activity in methanolic extracts of medicinal plants

**DOI:** 10.1186/1472-6882-12-3

**Published:** 2012-01-13

**Authors:** Leon IC Tang, Anna PK Ling, Rhun Y Koh, Soi M Chye, Kenny GL Voon

**Affiliations:** 1 School of Medical Sciences, Faculty of Medicine and Health, International Medical University, Bukit Jalil, Kuala Lumpur, Malaysia; 2 Department of Human Biology, Faculty of Medicine and Health, International Medical University, Bukit Jalil, Kuala Lumpur, Malaysia; 3 Department of Pathology, Faculty of Medicine and Health, International Medical University, Bukit Jalil, Kuala Lumpur, Malaysia

## Abstract

**Background:**

Dengue fever regardless of its serotypes has been the most prevalent arthropod-borne viral diseases among the world population. The development of a dengue vaccine is complicated by the antibody-dependent enhancement effect. Thus, the development of a plant-based antiviral preparation promises a more potential alternative in combating dengue disease.

**Methods:**

Present studies investigated the antiviral effects of standardised methanolic extracts of *Andrographis paniculata, Citrus limon, Cymbopogon citratus, Momordica charantia, Ocimum sanctum *and *Pelargonium citrosum *on dengue virus serotype 1 (DENV-1).

**Results:**

*O. sanctum *contained 88.6% of total flavonoids content, an amount that was the highest among all the six plants tested while the least was detected in *M. charantia*. In this study, the maximum non-toxic dose (MNTD) of the six medicinal plants was determined by testing the methanolic extracts against Vero E6 cells *in vitro*. Studies also determined that the MNTD of methanolic extract was in the decreasing order of *M. charantia *>*C. limon *>*P. citrosum, O. sanctum *>*A. paniculata *>*C. citratus*. Antiviral assay based on cytopathic effects (CPE) denoted by degree of inhibition upon treating DENV1-infected Vero E6 cells with MNTD of six medicinal plants showed that *A. paniculata *has the most antiviral inhibitory effects followed by *M. charantia*. These results were further verified with an *in vitro *inhibition assay using MTT, in which 113.0% and 98.0% of cell viability were recorded as opposed to 44.6% in DENV-1 infected cells. Although methanolic extracts of *O. sanctum *and *C. citratus *showed slight inhibition effect based on CPE, a significant inhibition was not reflected in MTT assay. Methanolic extracts of *C. limon *and *P. citrosum *did not prevent cytopathic effects or cell death from DENV-1.

**Conclusions:**

The methanol extracts of *A. paniculata *and *M. charantia *possess the ability of inhibiting the activity of DENV-1 in *in vitro *assays. Both of these plants are worth to be further investigated and might be advantageous as an alternative for dengue treatment.

## Background

Dengue disease regardless of its serotypes is transmitted from person to person by *Aedes aegypti *and *Aedes albopictus *mosquitoes in the domestic environment [1]. In the recent decade, dengue has re-emerged and with it being endemic in more than 110 countries, it has been the most prevalent arthropod-borne viral diseases in terms of morbidity and mortality [2]. Two fifths of the world populations are at risk, estimating around 100 million of dengue fever infections, 2.1 million cases of dengue hemorrhagic fever and 200 thousand deaths worldwide are caused by dengue every year. Despite extremely high rates of dengue for decades, Southeast Asia region still recorded an increase of 67% from 1985-1989 to 2002-2006 [3]. Similarly, a total of 4.6-fold increase in dengue cases has also been reported in America over the three decades [3].

Dengue appears in two forms; the first classic dengue fever with symptoms that range from mild fever to high fever with retro-orbital pain, severe headaches, maculopapular rashes, muscle and joint pain. The other more severe form, Dengue Hemorrhagic Fever (DHF) and Dengue Shock Syndrome (DSS) may present with abdominal bleeding, hemorrhage and circulatory failure, which is fatal if without prompt and proper management [4]. There are four serologic types of dengue virus, DENV-1, -2, -3 and -4. A primary infection with any of the four serotypes results in a lifelong immunity to that serotype, and temporary immunity to the others. However, this temporary immunity usually wanes after 6 months, at which point an individual is susceptible to the other three DENV serotypes [5]. The primary infection is most often asymptomatic, but sequential infections in the presence of heterologous dengue antibodies often leads to a more severe secondary infection causing DHF or DSS. Murrel et al. [5] attributed this to the antibody-dependent enhancement (ADE) effect. Studies on the outbreaks in endemic areas, such as South East Asia revealed that a primary infection with DENV-1 or DENV-3 frequently resulted in a more severe disease than if DENV-2 or DEV-4 were the primary infection [6].

With the rapid expansion of dengue disease in most tropical and subtropical areas of the world, it is crucial to develop effective prevention and control measures, including antiviral drugs and vaccines against dengue disease. However, the development of a dengue vaccine is complicated by the ADE effect, which happens when a child experiences a second dengue infection with a different serotype from the previous infection, causing DHF/DSS. The vaccine must not only protect against all four dengue serotypes but also must avoid inducing the ADE effect. Early studies have shown that extract from different parts of plants could provide good antiviral results as compared to their synthetic analogues [7]. As such, the development of a plant-based antiviral preparation promises a more potential alternative in combating dengue disease. Over the years, the World Health Organization (WHO) advocated that countries should interact with traditional medicine with a view to identify and exploit aspects that provide safe and effective remedies for ailments of antiviral. In addition, there are still no antiviral drugs being tested against dengue disease in any clinical trials. As such, present studies aimed to screen and determine the anti-dengue activity of methanolic extracts of *Andrographis paniculata *(Burm. f.) Nees*, Citrus limon *(L.) Burm. f., *Cymbopogon citratus *(DC.) Stapf*, Momordica charantia *L*, Ocimum sanctum *L. and *Pelargonium citrosum*, which have been used as folk medicine for dengue.

## Methods

### Plant materials and extraction

*Andrographis paniculata, Ocimum sanctum, Pelargonium citrosum *and *Cymbopogon citratus *plants were collected from Sungai Buluh, Selangor, Malaysia while *Citrus limon *and *Momordica charantia *were collected from Sibu, Sarawak and Sri Serdang, Selangor, respectively. The botanical identities of each plant were determined and authenticated by taxonomist from Institute of Bioscience, University Putra Malaysia. After the taxonomy identification, the plants were washed and cleaned prior to air drying at room temperature for one week. The whole aerial body of *A. paniculata, O. sanctum, P. citrosum *and *C. citratus *was blended and used. As for *C. limon *and *M. charantia*, the roots and entire fruits were utilised, respectively. Upon drying, 100 g of the plant materials were ground to powder and extracted consecutively with 500 mL of absolute methanol (Fisher Scientific, UK) for three to four days in dark condition. The resulting suspension was filtered and evaporated under reduced pressure at 50°C until dryness.

### Standardisation of extracts

Standardisation of extracts was carried out based on the total flavanoids content of the extracts measured using aluminium chloride colourimetric assay as described by Pourmorad et al. [8]. For this assay, 0.1 mg of extract was dissolved in 1 mL of methanol. The solution was then mixed with 0.1 mLof 10% (v/v) aluminium chloride (Sigma-Aldrich, USA), 0.1 mL of 1 M potassium acetate (Sigma-Aldrich, USA) and 2.8 mL of distilled water. After 30 minutes of incubation at room temperature, the absorbance reading of the reaction mixture was measured at 415 nm using spectrophotometer (UV-Vis Spectrophotometer, Japan) with methanol as blank. A standard curve was constructed using quercetin (Sigma-Aldrich, USA) as the standard at the concentrations ranging from 50 to 200 μg/mL. Total flavonoids content of the extracts was then compared with the standard curve, expressed in mg quercetin equivalents per 100 g dry weight (mg QE/100 g DW) and further calculated as % of total flavonoids content.

### Preparation of extracts

For the cytotoxicity and antiviral assays, a stock solution was prepared by dissolving 2.0 g of extract in 100 mL of dimethyl sulfoxide (DMSO) (Sigma Aldrich, USA). The stock solution was filter sterilised (0.20 μm pore, Minisart) and further diluted with culture medium to the desired concentration for the assays.

### Preparation of medium

Powdered Dulbecco's Modified Eagle's Medium (DMEM) (GIBCO, UK) was used in this study. A total of 3.7 g of sodium bicarbonate was added, dissolved with 1 L of ultrapure water and the pH of the medium was adjusted to 7.0. The medium was then filter sterilised using 0.22 μm PES membrane filter (TPP, Switzerland) under vacuum condition.

### Maintenance of Vero E6 cells

The cryopreserved Vero E6 cells were rapidly thawed at 37°C in a water bath. Cells were transferred carefully to 25 cm^2 ^tissue culture flasks (Corning, USA) containing 4 mL of DMEM with 10% of Fetal Bovine Serum (FBS) (GIBCO, South America). Cells were then incubated at 37°C with 5% CO_2 _for 2 to 3 days until confluent.

At 80-100% confluency, the cells were subcultured. The subculturing process was initiated by removing the used medium followed by rinsing the cells twice with Phosphate buffer solution (PBS) (MP Biomedical, France). Then, 1 mL of 0.25% trypsin-EDTA (GIBCO, Canada) was added and incubated for 5 to 10 minutes at 37°C. After trypsinisation, 1 mL of fresh medium with 10% FBS was added and mixed. The mixture was then centrifuged for 10 minutes at 1500 rpm. The supernatant was discarded while the pellet was resuspended with 2 mL of fresh medium and re-distributed into new tissue culture flask for further maintenance.

### Preparation of virus stock

The serotype of dengue virus obtained from Makmal Kesihatan Awam Kebangsaan (MKAM), Malaysia was confirmed through Polymerase Chain Reaction (PCR) and sequencing. The PCR was conducted based on the protocols described by Liu et al. [9]. In brief, the nucleotide sequence (~400 bp) of the dengue virus was amplified using the primers, AD3 5' CTGATTTCCATCCCGTA 3' and AD4 5' CATATGGGTTATTGGATAGA 3'. The PCR products were then sequenced. The sequencing results were compared with the library sequences using Basic Local Alignment Search Tool (BLAST). Thus, the dengue virus obtained was confirmed to be of serotype 1 (DENV-1).

The stock of dengue virus was obtained by adding 1 mL of DENV-1 to confluent Vero E6 cells and gently shaken for 1 hour to maximise the viral adsorption to the cells. After which, 4 mL of fresh medium with 10% FBS was added prior to incubation at 37°C with 5% CO_2 _for six to seven days. Supernatant was then collected and stored at -80°C. These were repeated for several times until adequate virus stock was collected.

### Determination of maximum non-toxic dose (MNTD)

The *in vitro *cytotoxicity assay was carried out on the extracts to determine the MNTD on Vero E6 cells. The cytotoxicity assay was initiated by seeding 1.5 × 10^4 ^cells into 96 well flat-bottom plates (Corning, USA). A blank control (medium only) and cell control (cells only) were also plated. The plate was then incubated in 5% CO_2 _humidified incubator (RS biotech, Galaxy S) for 24 hours. After 24 hours, the cells were treated with diluted stock extract at the concentrations ranging from 0.05 to 2.5 mg/mL, and later further incubated at 37°C with 5% CO_2_.

After 96 hours, 20 μL of 3-(4,5-Dimethylthiazol-2-yl)-2,5-diphenyltetrazolium bromide (MTT) salt solution was added into each well and incubated for 4 hours. After 4 hours, the supernatant of the well was carefully removed. A total of 100 μL of DMSO was added followed by continuous shaking for 10 minutes. The absorbance reading of each well was measured using microplate reader (Tecan, Austria) at 570 nm. The percentage of cell viability and toxicity was further determined based on the absorbance readings.

### Determination of median tissue culture infective dose (TCID_50_)

A total of 1.5 × 10^4 ^cells/well were seeded into 96-well plate and incubated at 37°C with 5% CO_2_. After 24 hours, a total volume of 100 μL of ten-fold serially diluted DENV-1 was inoculated into each well with 10 replicates for each dilution. Plates were further incubated at 37°C for 5 days, after which cytopathic effect (CPE) was observed microscopically under inverted microscope. To determine the TCID_50 _based on Karber method [10], the presence of CPE in each well was marked as '+', while its absence was marked as '-'. The proportion of wells with CPE in each serially diluted DENV-1 was calculated and the TCID_50 _was estimated using the formula 'Log TCID_50 _= L - d (s - 0.5)', whereby L = lowest dilution factor; d = difference between dilution steps; s = sum of proportion. The value of TCID_50 _determined was applied in the antiviral assay.

### *In vitro *antiviral assay

The *in vitro *antiviral assay was initiated by seeding 1.5 × 10^4 ^cells/well into each well of 96-well plate, and left to incubate at 37°C with 5% CO_2 _for 24 hours. After 24 hours, the medium was removed and 100 μL of each plant extract at its MNTD was added. After incubating for 1 hour, 100 μL of DENV-1 at its TCID_50 _was added. The assay was also conducted with the controls, which included the cells alone, cells treated with extracts and cells with DENV-1. The 96-well plate was further incubated for five days, after which the CPE of the cells were observed under inverted microscope on a daily basis. The antiviral effect of each plant extract was assessed using a grading system as described by Kudi and Myint [11], whereby the degree of CPE inhibition upon treatment was marked at the following order: '++++' represented a total inhibition, '+++' for 75% inhibition, '++' for 50% inhibition, '+' for less than 50% inhibition, '-' as no inhibition. The antiviral assay for each plant extract was conducted in five replicates and repeated twice.

The potency of these extracts on DENV-1 inhibition were also further examined using MTT assay, in which the percentage of viability of cells alone, cells infected with DENV-1, and infected cells treated with extracts at MNTD were measured and compared. Statistical difference between treatments and positive control was analysed using unpaired *t*-test at *p *value less than 0.05 (*p *< 0.05). The test was performed using GraphPad InStat version 3.0.

## Results and discussion

### Standardisation of extracts

In this study, the methanol extracts of the six plants were standardised based on the total flavonoids content determined through aluminium chloride colourimetric assay.

Earlier observations confirmed that flavonoids in plants and their derivatives possess antiviral activity [12,13]. Thus, in this study, the methanolic extracts were standardised based on total flavonoids content prior to antiviral assay. Present study reported that total flavonoids content enfolded a total of 87% of the methanolic extract of *O. sanctum *(Table 1). Medicinally importance flavonoids identified in *O. sanctum *were orientin, vicenin and luteolin [14,15]. Orientin and vicenin are reported to have radical scavenging activity [16] while lutoelin is known to be anti-inflammatory agent and have therapeutic action against multiple sclerosis [17,18]. *M. charantia *possessed a total flavonoid content of 21.7%, of which some of them could be luteolin, kampherol and quercetin [19]. A study by Lin & Tang[20] obtained a total flavonoids content that was slightly lower, at a value of 15.0%. This could be due to the age and geographical locations of the plants as well as the temperature used during extraction [21]. In this study, *A. paniculata *is another plant that recorded low amount of total flavonoids content. In *A. paniculata*, some flavonoids that have been isolated were 7-O-methylwogonin, apigenin, onysilin and 3,4-dicaffeoylquinic acid, which are anti-atherosclerotic [22].

**Table 1:** The total flavonoids content of six medicinal plants as determined through aluminium chloride colourimetric assay.

Plant	Total flavonoids content (%)
*Momordica charantia*	21.7 ± 10.9
*Andrographis paniculata*	24.3 ± 3.0
*Citrus limon*	32.6 ± 6.7
*Cymbopogon citrates*	35.2 ± 10.1
*Pelargonium citrosum*	61.1 ± 10.2
*Ocimum sanctum*	88.6 ± 21.4

Meanwhile, *C. limon *possessed an average amount of total flavonoids content (33%) among the six plants studied. *C. limon *is a good source of flavonoids such as hesperidin, diosmin and eriocitrin [23]. Root bark from the *Citrus *spp. possessed the flavonoids; citrunobin, citflavanone and lonchocarpol-A [24]. Meanwhile, *C. citratus *showed an average total flavonoids content of 34.2%. Reported flavonoids isolated from *C. citratus *were luteolin, apigenin, homoorintine flavanoides and its 2-O rhamnosil-limborientino [25,26]. Among the six plants studied, *P. citrosum *recorded a high percentage of total flavonoids content. The reason underlying high flavonoids content in methanolic extracts of *P. citrosum *is yet to be known as there is still a lack of investigation on the flavonoid compounds in this plant. Up to now, apart from the report on the presence of small amount of tannins and coumarins [27], most of the studies on *P. citrosum *have been focused on its main constituent, essential oils.

### Determination of maximum non-toxic dose (MNTD)

**Figure 1:**
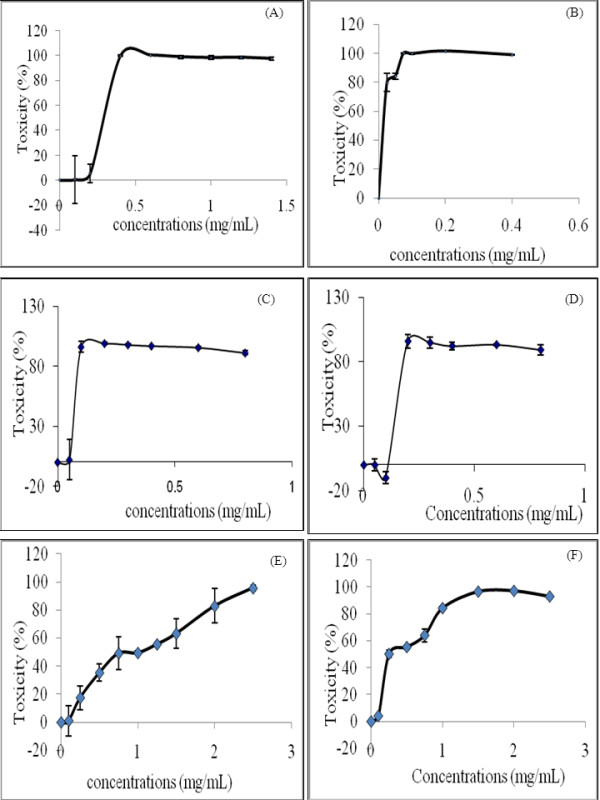
**The percentage of toxicity of methanolic extracts of six plants on Vero E6 cells after incubated *in vitro *for 96 hours**. (A) *Momordica charantia *(B) *Cymbopogon citratus *(C) *Andrographis paniculata *(D) *Citrus limon *(E) *Pelargonium citrosum *(F) *Ocimum sanctum*. The data shown are means ± S.D. of two independent experiments performed in triplicates.

Prior to evaluating the anti-dengue properties of six medicinal plants, their methanolic extracts were subjected to toxicity studies in order to determine the maximal dose, which could be non-toxic to the cells. In this study, the MNTD of the six medicinal plants was determined by testing the methanolic extracts against Vero E6 cells *in vitro*. The studies were initiated by using ten-fold serially diluted methanolic extracts of each plant followed by further optimisation in order to achieve a specific cytotoxic concentration. The MNTD of each plant obtained through the optimization steps were presented in Figure 1.

In general, the cytotoxicity studies discovered that MNTD for methanolic extract of *M. charantia *was 0.20 mg/mL, a value which was the highest among all the six plants examined (Figure 1A). In contrast, MNTD of *C. citratus *was the lowest, that was at a minute concentration of 0.001 mg/mL (Figure 1B) whilst the plant from Acanthaceae family, *A. paniculata *recorded the second lowest MNTD (0.050 mg/mL) (Figure 1C). Studies also revealed that there was no significant difference in MNTD values for *C. limon, P. citrosum *and *O. sanctum *(Figures 1D-F), in which all the three extracts attained MNTD that was two times higher as compared to *A. paniculata*. In terms of the pattern of cytotoxity, the extracts of *C. citratus, A. paniculata, C. limon *and *M. charantia *showed steep rises of cytotoxicity and reached the toxic limits over a narrow concentration range. Among all the plants, *C. citratus *showed maximum cytotoxicity at 0.075 mg/mL, indicating that it is the most cytotoxic plant. Meanwhile, *P. citrosum *and *O. sanctum *exhibited slow rises in cytotoxicity over a wide range of concentrations.

Cytotoxic studies are essential for estimating the therapeutic range of plant extract on mammalian cells. In the present studies, both *O. sanctum *and *P. citrosum *have the highest cytotoxic values (1.5 mg/mL and 2.5 mg/mL, respectively). A high cytotoxic value indicated that these two plants were less toxic as compared to the other four plants studied. As determined in this study, these two plants also recorded the highest amount of total flavonoids content. Hence, it is anticipated that the flavonoids present in the methanolic extract might have contributed to a high cytotoxic concentration. Flavanoids have been reported to possess antioxidative activities, such as to inhibit lipid peroxidation, chelate redox-active metals and inhibit free-radical mediated events and even increasing resistance to DNA strand breakage [28,29]. These protect the cells by scavenging of free radicals that cause oxidative stress and can also induce changes in membrane to reduce membrane lipid and protein oxidation. Oteiza et al. [30] suggested that the flavonoids interact at the surface of bilayers to reduce the access of harmful oxidants, thus protecting the structure and function of membranes.

### *In vitro *antiviral assay

In order to screen the anti-dengue properties of methanolic extracts of the six medicinal plants, *in vitro *antiviral assay was conducted. The antiviral assay was initiated using the MNTD of each plant extract against TCID_50_. Prior to the qualitative observation of antiviral assay, a grading system was developed according to Kudi and Myint [11]. This system denoted the degree of inhibition seen under inverted microscope using the '+' symbol. The more '+' symbols signified a higher percentage of inhibition was observed whereas the '-'symbol indicated no inhibition or widespread cell death. Thus, '++++' represented a total inhibition, '+++' for 75% inhibition, '++' for 50% inhibition, '+' for less than 50% inhibition and '-' as no inhibition was observed upon the treatment with methanolic extracts.

**Figure 2:**
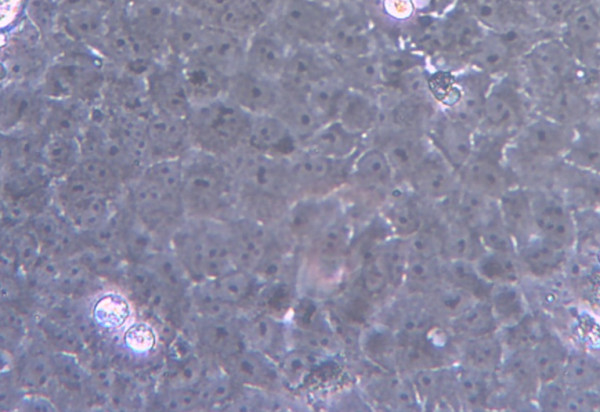
**A monolayer sheet of uninfected Vero E6 cells**. The normal cells were polygonal with well defined, black nuclei in the centre. The cytoplasm was shaded grey with the cell membranes were clearly demarcated with white lines surrounding the cells. The cells were viewed under inverted microscope at 200× magnification.

The Vero E6 cells grown in the tissue culture flasks with DMEM supplemented with 10% FBS would form a monolayer sheet of cells. The morphology of Vero E6 cells were clearly visualised using an inverted light microscope. Under 200× magnification, the uninfected Vero E6 cells were polygonal in shape with well defined, black nuclei in the centre. The cytoplasm was shaded grey but the cell membranes were clearly demarcated with white lines surrounding the cells as shown in Figure 2.

**Figure 3:**
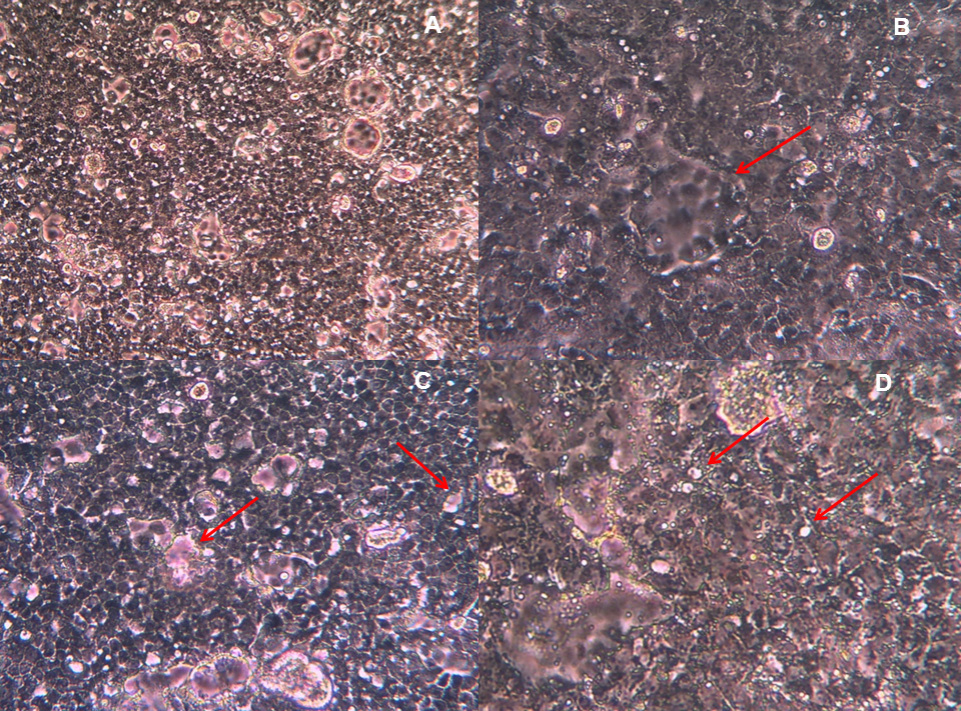
**Various cytopathic effects observed in DENV-1-infected Vero E6 cells after 7 days post infection**. (A) The monolayer sheet of cells at 100× magnification appeared to be congested and haphazard. (B) A typical syncythial giant cell formation or syncytia resulting from the fusion of a few cells infected with DENV-1 viewed at 200× magnification. The morphological changes appeared to be group of cells without a clear white demarcation of cell membrane. The cells located at the perimeter of the syncytia were observed clearly. (C) Areas of cell death or lysis are pointed out by the arrows. These small areas were clear of any cells and reflected the colour of the pink medium. (D) A higher magnification image (400×) showing white dots called blebs.

Apart from the uninfected cells, the DENV1-infected Vero E6 cells were also observed under inverted microscope. The various cytopathic effects (CPE) after 7 days post infection were examined at 100×, 200× and 400× magnifications as some CPE could be better viewed at different magnifications. From the observation, it was clearly shown that DENV1-infected cells showed CPE, which refers to the structural changes of the host cell due to viral infection. Generally, in comparison to the uninfected monolayer sheet of Vero E6 cells, DENV1-infected cells appeared to have a congested and haphazard pattern (Figure 3A). Cytopathic effects included syncytia formation (Figure 3B) and cell lysis (Figure 3C). Syncytia formation could be due to viral fusion proteins being transported to the cell surface membranes, which later fused with neighbouring cells. In addition, blebbing of cells (Figure 3D), which referred to small detached remnants of apoptotic bodies that have undergone programmed cell death could also be observed upon DENV-1 infection.

**Figure 4:**
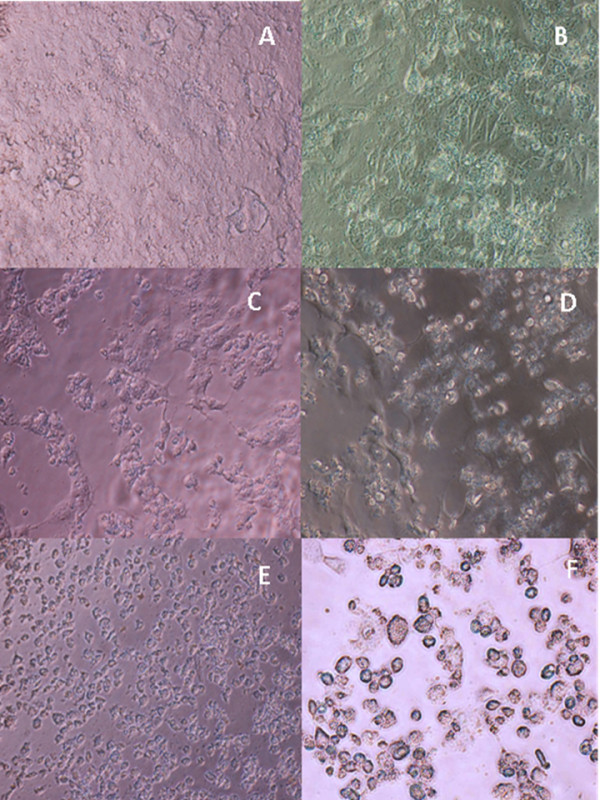
**Morphological changes of DENV-1-infected Vero E6 cells treated with methanolic extracts of six medicinal plants at 7 days post infection**. All the cells were viewed under inverted microscope at 200× magnification. (A) *A. paniculata *with75% of inhibition. (B) *M. charantia *with 50% of inhibition. (C) *O. sanctum *with less than 50% inhibition. (D) *C. citratus *with less than 50% inhibition (E) *C. limon *with no inhibition. Only cell fragments and debris were seen (F*) P. citrosum *with no inhibition. Granulation and cell densing were observed. The CPE for each treatment was compared with those of negative and positive controls shown in Figures 2 and 3, respectively.

Table 2 depicts the degree of inhibition on DENV-1 by the six medicinal plants. Among the six plants studied, *A. paniculata *showed the most antiviral inhibitory effects, that was 75% of inhibition. The methanolic extract of *A. paniculata *was able to maintain most of the normal cell morphologies without causing much CPE to the DENV-1 infected cells. The monolayer sheet of cells still remained normal with low amount of cells death or lysis as well as low percentage of CPE (Figure 4A). In addition to *A. paniculata, M. charantia *also exhibited moderate antiviral effects. Viable normal cells were observable in the background. Nevertheless, around 50% of the cells within the treatment showed CPE, which was indicated by the changes of shape of cells to spindle shaped (Figure 4B). The potency of these two extracts in inhibiting replication of DENV-1 was further confirmed through MTT assay, whereby the viability of cells treated with both *A. paniculata *and *M. charantia *extracts were not significantly affected upon infection with DENV-1 (Table 3).

**Table 2:** Antiviral assay based on cytopathic effects denoted by degree of inhibition upon treating the DENV1-infected Vero E6 cells with maximum non-toxic dose of six medicinal plants.

Plant species	Degree of inhibition
*Andrographis paniculata*	+++
*Momordica charantia*	++
*Cymbopogn citratus*	+
*Ocimum sanctum*	+
*Citrus limon*	-
*Pelargonium citrosum*	-

+++ 75% inhibition, ++ 50% inhibition, + < 50% inhibition, - no inhibition

**Table 3:** The percentage of cell viability as measured by MTT assay upon treating the DENV1-infected Vero E6 cells with maximum non-toxic dose of six medicinal plants.

Treatments	Cell viability (%)
Control (Untreated cells)	100.00 ± 10.50*
Positive control (Cells infected with DENV-1)	44.60 ± 4.42
*Andrographis paniculata*	113.00 ± 4.65*
*Momordica charantia*	98.00 ± 8.69*
*Cymbopogn citrates*	32.00 ± 6.93
*Ocimum sanctum*	48.00 ± 7.16
*Citrus limon*	52.00 ± 7.54
*Pelargonium citrosum*	41.40 ± 2.75

The data shown are mean ± S.D. '*' denotes significant difference at *P *< 0.05, compared to the positive control using unpaired *t*-test, performed by GraphPad Instat version 3.0.

Current preliminary screening study based on CPE also revealed that methanolic extracts of *O. sanctum *and *C. citratus *showed a low inhibition (less than 50%) against DENV-1. Observation of CPE under the inverted microscope showed the presence of fragmented cells and debris (Figures 4C and 4D). Nevertheless, this was not in accordance with the MTT assay conducted as only 48.0% and 32.0% of the cells treated with *O. sanctum *and *C. citrates *survived upon DENV-1 infection, respectively. The values recorded were not much differed from 44.6% achieved in the positive control that infected with DENV-1 (Table 3). The variation in findings based on CPE and MTT assay could be due to the fact that these extracts were able to inhibit the cytopatic effect without inhibiting the virus replication in Vero E6 cells. Among the six plant extracts tested, *C. limon *and *P. citrosum *did not prevent CPE or cell death from the effects of DENV-1. The normal polygonal-shaped cells were not visible followed by widespread of cell death, indicated by the amount of debris strewn across Figures 4E and 4F. Furthermore, cells treated with *P. citrosum *showed granulation and cell densing (pyknosis).

*A. paniculata, C. limon, C. citratus, M. charantia, O. sanctum *and *P. citrosum *have been traditionally known to possess antiviral activity. Out of the six plants tested, *C. citratus, O. sanctum, P. citrosum *and *C. limon *showed no anti-dengue activity even though some of their methanolic extracts exhibited high total flavonoids content. One of the possible explanations to this non-inhibitory effect could be that there lies a relationship between the structure of the flavonoids and antiviral activity as described by Sanchez et al. [12]. It was found in their comparative study that flavonoids with no prenyl side-chain totally lacked anti-dengue activity. *P. citrosum *and *C. limon *extracts showed obvious cell death when treated with DENV-1 infected control cells. A possible explanation to this result could be that the compounds present in these plants could have affected cell proliferation instead of virus replication [12], thus, preventing the cell sheet from regrowing after DENV-1 infected cell death.

In this preliminary screening study for anti-dengue agent, methanolic extract of *A. paniculata *and *M. charantia *was found to have high potential to be an anti-dengue agent, particularly towards DENV-1 serotype. *A. paniculata *extract showed a total of 75% of inhibition while *M. charantia *showed approximately 50% of inhibition on CPE upon infection with DENV-1. These findings suggested that the anti-dengue activity might be owing to the presence of flavonoid compounds or other compounds such as terpenes and polyphenols that were extractable by methanol. Various flavonoid compounds have been documented to possess antiviral properties. For instance, *M. charantia *that was reported to show anti-HSV-1, polio type 1, parainfluenza and respiratory syncytial virus activities [21] was found to contain flavonoids such as lutelin, kampherol and quercetin [19]. Flavonoids are able to inhibit viruses by inhibiting different pathways. Chang et al [31] disclosed that dehydroandrographolide succinic acid monoester (DASM) could interfere with cell fusion, thus preventing HIV from entering the cell. Alternately, certain flavonoids with potent anti-HIV effects are capable of inhibiting virus associated reverse transcriptase and even cellular DNA or RNA polymerase [32].

Even though flavonoid compounds have been the main constituent of interest in search of anti-dengue agent, the role of diterpenes such as andrographolide, 14-deoxyandrographolide and 14-deoxy-11,12-didehydroandrographolide present in methanolic extract of *A. paniculata *[33] against DENV-1 should not be neglected. Andrographolide, and 14-deoxy-11,12-didehydroandrographolide have been reported to exhibit anti-HIV activity [34]. Similarly, methanolic extract of *M. charantia *was found to contain terpenes, saponins and steroids [35,36]. In a related study, the methanolic extract of *M. charantia *was presumed to contain the major compound such as unglycosides terpenes [37]. However, there is a lack of literature to support that unglycoside terpenes cause any antiviral activity.

There are two probable pathways that could be the mode of antiviral action: 1) interfere with viral adsorption, and 2) inhibit viral replication. Schitzler et al. [38] noted that pre-treatment of the cells might give the cells protective effects against viruses by preventing viral adsorption. Furthermore, the dengue virus E protein is a potential target of inhibition due to its principal role in cell membrane adhesion [39]. As such, there is a possibility that the extract supplied might have blocked the E protein. Alternatively, the inhibitive event could have also occurred during viral replication. One stage of inhibition of viral replication by flavonoids was found using flavonoid SP-303, which showed a mode of antiviral action on DNA and RNA synthesis [32]. Another stage of inhibition of viral replication might be by blocking certain enzymes, such as an antiviral compound (NITD-982) that inhibit an enzyme needed for pyrimidine biosynthesis [40]. Non-structural proteins are also potential targets of inhibition. For example, a study on STAT2, a component of type-1 interferon signalling pathway that is targeted for degradation once nNS5 is bound to it. The study showed that mouse STAT2 could restrict early dengue virus replication *in vivo *[41].

## Conclusions

Studies on *Andrographis paniculata *and *Momordica charantia *that showed anti-dengue properties should be further conducted extensively. Isolation, purification and characterisation of the active compounds in order to discover the potential anti-dengue compounds should be carried out. Furthermore, investigations into the mode of action of anti-dengue activities by the active compounds can be done to provide more insight into inhibition of dengue adsorption and replication.

## List of abbreviations

CPE: cytopathic effects; DENV-1: Dengue virus serotype 1; DMEM: Dulbecco's Modified Eagle's Medium; DMSO: dimethyl sulfoxide; FBS: Fetal Bovine Serum; MNTD: maximum non-toxic dose; TCID_50_: median tissue culture infective dose

## Competing interests

The authors declare that they have no competing interests.

## Authors' contributions

APKL participated in the design and coordination of the study, carried out the extraction and standardization studies and edited the manuscript. LICT carried out the extraction and standardization studies, participated in the cytotoxicity and anti-viral assay and drafted the manuscript. RYK helped in the design of cytotoxicity and antiviral assay. SMC participated in the design and anti-viral assay. KGLV participated in the PCR and sequencing studies. All authors read and approved the final manuscript.

## Pre-publication history

The pre-publication history for this paper can be accessed here:

http://www.biomedcentral.com/1472-6882/12/3/prepub
